# Quadruple-junction lattice coherency and phase separation in a binary-phase system

**DOI:** 10.1038/ncomms9252

**Published:** 2015-09-08

**Authors:** Sung-Yoon Chung, Si-Young Choi, Jin-Gyu Kim, Young-Min Kim

**Affiliations:** 1 Graduate School of EEWS, Korea Advanced Institute of Science and Technology (KAIST), 291 Daehak-ro, Yuseong-gu, Daejeon 34141, Korea; 2 Korea Institute of Materials Science, 797 Changwon-daero, Seongsan-gu, Changwon 51508, Korea; 3 Korea Basic Science Institute, 169-148 Gwahak-ro, Yuseong-gu, Daejeon 34133, Korea

## Abstract

If each phase has an identical crystal structure and small misfit in the lattice parameters in a binary-phase crystalline system, coherent phase boundaries usually form during separation. Although there have been numerous studies on the effect of coherency elastic energy, no attempt has been made to demonstrate how the phase-separation behaviour varies when multiple interfaces meet at a junction. Here we show that a comprehensively different phase-separation morphology is induced, to release the high coherency strain confined to quadruple junctions. High-temperature *in-situ* transmission electron microscopy reveals that phase boundaries with a new crystallographic orientation emerge over twinned crystals to provide strain relaxation at quadruple junctions. The high coherency strain and the formation of different phase boundaries can be understood in terms of the force equilibrium between interface tensions at a junction point. Visualizing the quadruple points at atomic resolution, our observations emphasize the impact of multiple junctions on the morphology evolution during phase separation.

## Abstract (web summary)

The overall physical properties of binary-phase systems ranging from metallic alloys to soft polymers are affected by phase-separation morphologies. Here, Chung *et al.* use atomic-resolution electron microscopy to demonstrate the influence of quadruple junctions on the morphology evolution during phase separation.

## Graphical abstract

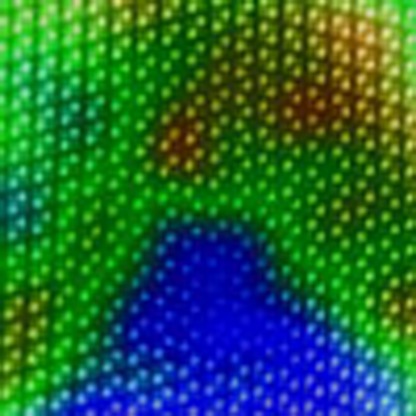

A wide spectrum of studies on how two phases separate in binary-phase systems can be found in the literature, ranging from the modulated structure in metallic alloys and oxides^1^,^2^,^3^,^4^,^5^,^6^ to a variety of peculiar separation morphologies in block-copolymer-based soft materials^7^,^8^. Among the numerous factors affecting the phase separation behaviour, elastic strain at interfaces between two phases plays a major role during microstructure evolution when two phases having similar crystal structures and lattice parameters construct coherent interfaces^1^,^9^. Following the milestone work by Cahn^10^,^11^ on spinodal decomposition, theoretically and experimentally notable results have been reported on the relationship between the coherency elastic strain and the phase-separation morphologies over the past five decades^1^,^3^,^9^,^12^,^13^,^14^,^15^,^16^.

Crystal twinning can take place without any crystal deformation by external shear stress, as extensively studied in various polycrystalline metals and alloys (annealing twins)^17^,^18^ and also as frequently observed in many oxide minerals such as spinels, feldspars and gypsums during crystal growth (growth twins)^19^,^20^,^21^. As these twins are two-dimensional planar interfaces, quadruple junctions form when phase boundaries intersect the twinning planes during phase separation. A key aspect that should be taken into account at the quadruple junctions is that four interfacial tensions from the phase boundaries and the twin interfaces should satisfy their force equilibrium. Therefore, a complex correlation between the force balance and the coherency elastic strain is anticipated when the lattice coherency is maintained at the quadruple junctions, and direct experimental observations of the quadruple junctions at atomic resolution and the resultant phase-separation morphologies with crystal twins are necessary to shed light on this correlation.

By using *in-situ* transmission electron microscopy (TEM) at elevated temperature, scanning TEM (STEM) and geometric phase analysis (GPA), here we experimentally demonstrate the unexpected impact of quadruple junctions on the final morphology between two phases. Our work highlights two notable features. One is that we successfully image quadruple intersections at the atomic level, directly visualizing significant bending of phase boundaries at a quadruple junction, to satisfy the force balance between interfacial tensions. The other feature is that crystal twinning, which involves twin boundaries, can be effectively used to induce completely new phase morphologies that are unachievable without twin boundaries and subsequent quadruple intersections. Although the present study focuses on a single material system, we emphasize that the correlation between the multiple-junction force equilibrium and the phase separation behaviour is generally applicable to any crystalline system having anisotropic coherent strain with small lattice misfits.

## Results

### Observation of two-phase separation

**Figure 1:**
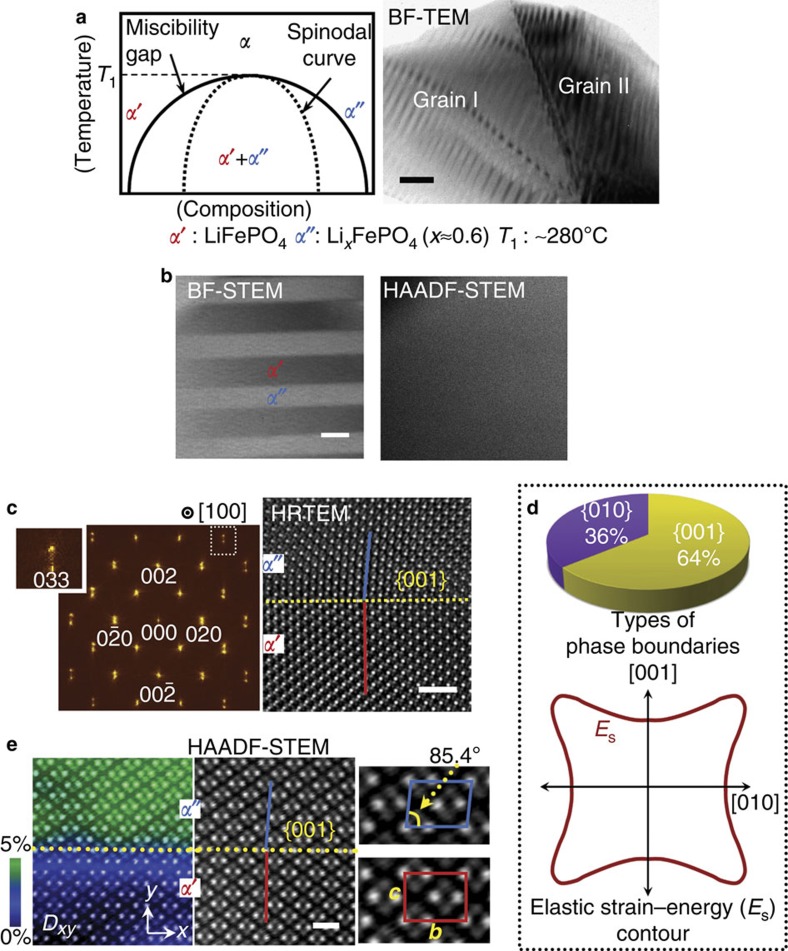
Two-phase separation and coherent phase boundaries with anisotropic strain energy. (**a**) A schematic phase diagram between phases *α*′ and *α*″ is illustrated, showing a miscibility dome below ∼280 °C. Two-phase stripe morphologies are observed in each grain of a polycrystalline sample (BF-TEM image). Scale bar, 100 nm. (**b**) Although the stripes can be imaged in BF-STEM, no contrast variation is observable in the HAADF-STEM image, owing to the identical [FePO_4_] framework with a difference in Li composition only. Scale bar, 20 nm. (**c**) A HRTEM image along with its FFT verifies the coherent {001} boundary between the two phases. Splitting of high-index Bragg reflections (for example, 033 in the enlargement) consistently supports the phase separation and the resultant boundary direction. Scale bar, 1 nm. (**d**) A pie diagram shows a statistical investigation results for phase boundaries from more than 40 grains. Based on these statistics, an anisotropic elastic strain-energy (*E*s) contour is qualitatively suggested, showing low *E*s orientations along the [001] and [010] directions. (**e**) The *α*′/*α*″ phases are readily discriminated in the HAADF-STEM image via the GPA. The relative shear displacement between the two phases due to the different interaxial angle (85.4°) of phase *α*″ is visualized on the *D*_*xy*_ map, indicating the position of the phase boundary (yellow line). Scale bar, 5 Å.

We selected an olivine-type Li_*x*_FePO_4_ as a model system having a miscibility gap. As depicted in the phase diagram in Fig. 1a, a solid-solution *α*-phase separates into lithium-rich LiFePO_4_ (*α*′) and lithium-deficient Li_0.6_FePO_4_ (*α*″) below ∼280 °C. The difference in lattice parameters of each end phase is <±1.5% (see Supplementary Note 1 and Supplementary Figs 1 and 2 for details regarding the crystal structure of olivine LiFePO_4_, the phase diagram and the Li_0.6_FePO_4_ phase)^22^,^23^. Therefore, as will be shown below, phase boundaries that form during separation are coherent. The bright-field (BF) TEM image in Fig. 1a reveals a typical two-phase lamellar structure observed in each grain from a polycrystalline sample with an initial composition of Li_0.75_FePO_4_ after furnace cooling for 8 h. The relative content of lithium is the major compositional difference between phases *α*′ and *α*″ under the same Li_*x*_FePO_4_ framework. Consequently, as presented in Fig. 1b, no distinct image feature appears in high-angle annular dark-field (HAADF) STEM, imaging of which is insensitive to the light elements^24^,^25^. In contrast, the BF-STEM image on the left-hand side exhibits a clear two-phase morphology due to the coherency strain across the boundaries. As in BF-TEM, a BF-STEM image is constructed similarly by the interference between the transmitted beam and the +*g* and −*g* Bragg reflections. The intensity should thus differ in each phase at the BF detector with a collection semiangle of 0−25 mrad, thereby resulting in distinct image contrast between the two phases in BF-STEM.

### Formation of coherent phase boundaries

From the minimum energy criterion, the preferential orientation of coherent interfaces between two phases is determined during separation so as to have the lowest elastic strain energy^1^,^16^. We examined phase boundaries in more than 40 grains from our polycrystalline sample to identify their crystallographic direction with minimum strain energy. Intriguingly, the orientation of the phase boundaries was not singular. Although the majority (64%) was {001} boundaries (Fig. 1c), some interfaces (36%) were parallel to the {010} plane, as illustrated in the pie diagram in Fig. 1d. In particular, most of the {010} phase boundaries were found in twinned grains. The high-resolution TEM (HRTEM) lattice images shown in Fig. 1c also verify that the {001} boundaries are completely coherent without any misfit dislocations. An additional supportive series of HRTEM images for phase boundaries, including the {010} boundaries, in various projections is provided in Supplementary Fig. 3. Based on these observations, a polar plot of the elastic strain energy (*E*_S_) in the [100] projection can be qualitatively suggested, as shown in Fig. 1d, indicating the two low *E*_S_ directions.

{001} phase boundaries were also examined in HAADF-STEM to observe their atomic structure in greater detail. As shown in the enlargements in Fig. 1e, the *b*–*c* interaxial angle is 85.4° in phase *α*″, in contrast to the perpendicularity in phase *α*′. This relative lattice displacement of phase *α*′′ with respect to the lattice of phase *α*′ (or vice versa) can be effectively visualized via GPA^26^,^27^,^28^. The colour map superimposed on the HAADF-STEM image in Fig. 1e shows a relative displacement (*D*_*xy*_) in the *xy* shear direction, readily positioning the *α*′(green)/*α*″(blue) phase boundary. The GPA is typically carried out using HRTEM images^26^ having better positional accuracy of lattice points than STEM images. However, recent advances in spherical aberration correction and mechanical stability in STEM enable utilization of the GPA with atomic-column resolved STEM images^27^,^28^,^29^,^30^, although the influence of image drift during acquisition should be taken into account. Details regarding utilization of a GPA to discriminate the *α*′/*α*″ phases in HAADF-STEM images are provided in Supplementary Note 2 along with Supplementary Fig. 4.

### *In-situ* TEM and HRTEM observations

**Figure 2:**
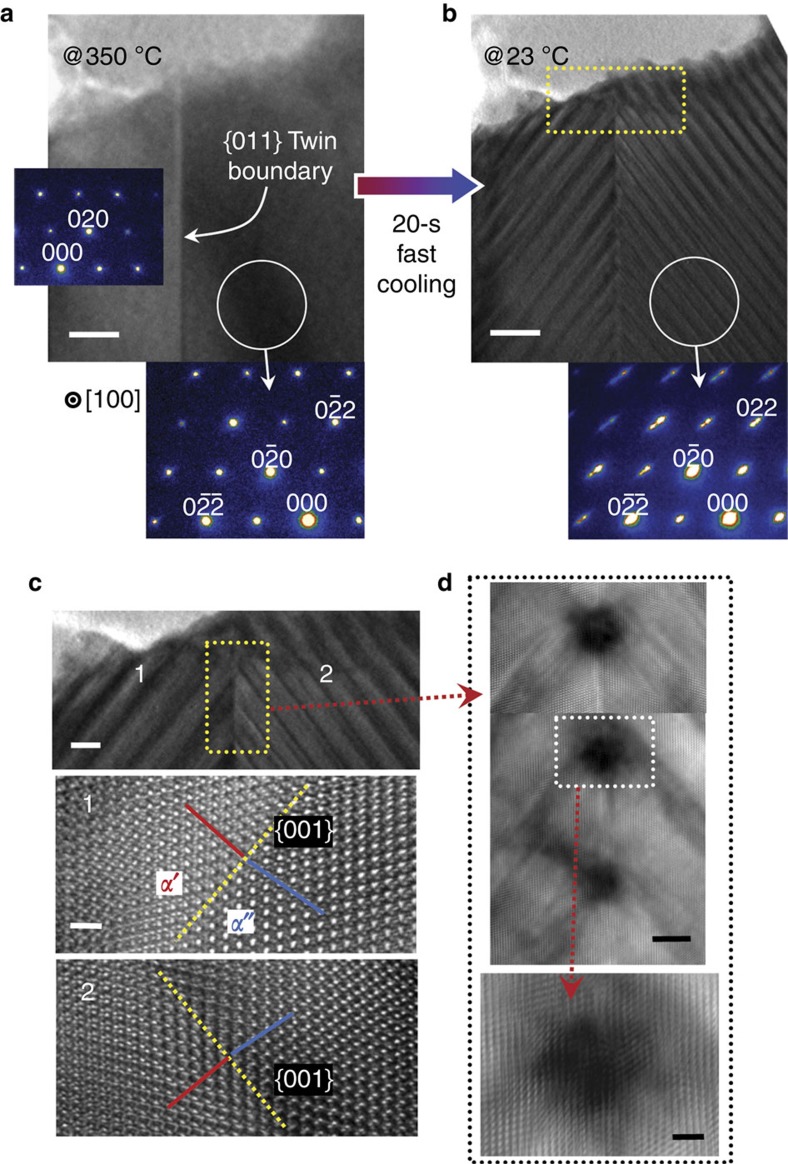
Observation of phase separation with a twin interface. (**a**,**b**) BF-TEM images of a grain containing a {011} twin boundary are shown. At 350 °C, this grain consists of a single phase. Two electron diffraction patterns in **a** confirm the twin relationship of both sides. Phase separation occurs when the grain is rapidly cooled to room temperature (**b**), as verified by the split Bragg spots in the diffraction pattern. Scale bar, 20 nm. (**c**) The magnified BF-TEM image for the region denoted by a yellow rectangle in **b** demonstrates a symmetrical Λ-shape morphology across a twin boundary. Two HRTEM images confirm the formation of {001} phase boundaries on both sides. Scale bar, 10 nm (upper panel) and 1 nm (middle panel). (**d**) Appearance of the periodic black image feature along the twin boundary is noted. An enlarged HRTEM image is provided for the local black contrast. Scale bar, 5 nm (upper panel) and 2 nm (lower panel).

We carried out *in-situ* TEM with a hot-stage heating holder, to monitor the microstructure evolution in twinned grains during phase separation in real time^31^. Each grain in a sample was verified to be a single *α*-phase at 350 °C above the miscibility gap during TEM observation. Figure 2a shows a single grain containing a {011} twin boundary (see Supplementary Note 3 together with Supplementary Fig. 5 for atomistic details of the twin boundary). When this grain was rapidly cooled to room temperature for 20 s (∼16 °C s^−1^ in cooling rate), two-phase separation in the form of modulated stripes immediately occurred (Fig. 2b). The distribution of the stripes is symmetrical across the {011} twin boundary, as can be seen in both Fig. 2b and the enlargement of Fig. 2c, resulting in a Λ-shape phase morphology. The two HRTEM images in Fig. 2c also show that all the phase boundaries at both sides are along the {001} plane. As already demonstrated in Cu-Ni-Fe alloys^3^, quenching a two-phase crystalline system far below the miscibility dome leads to coherent spinodal decomposition with minimum elastic strain. This rapid cooling experiment thus directly proves that the {001} phase boundaries are of the lowest coherency strain energy in our system.

One of the notable aspects observed in the twinned grain after phase separation is the periodic appearance of a strong black contrast at the twin boundary. The upper panel of Fig. 2d shows a magnified HRTEM image for the region denoted by a yellow rectangle in Fig. 2c, revealing the presence of black image features at an interval of ∼15 nm. These strong contrasts in the HRTEM image appear to be analogous to the typical image features usually ascribed to local strain fields, as demonstrated in other oxides and metals^32^,^33^. Considering that the two-phase separation in this system entails coherent phase boundaries, it is likely to be that remarkably high strain energy has been induced when *α*′/*α*″ phase boundaries intersect the twin interface during phase separation. The enlargement (lower panel) in Fig. 2d also provides a supportive image showing the strain contrast confined at the intersection.

### Atomic-scale STEM imaging and GPA

**Figure 3:**
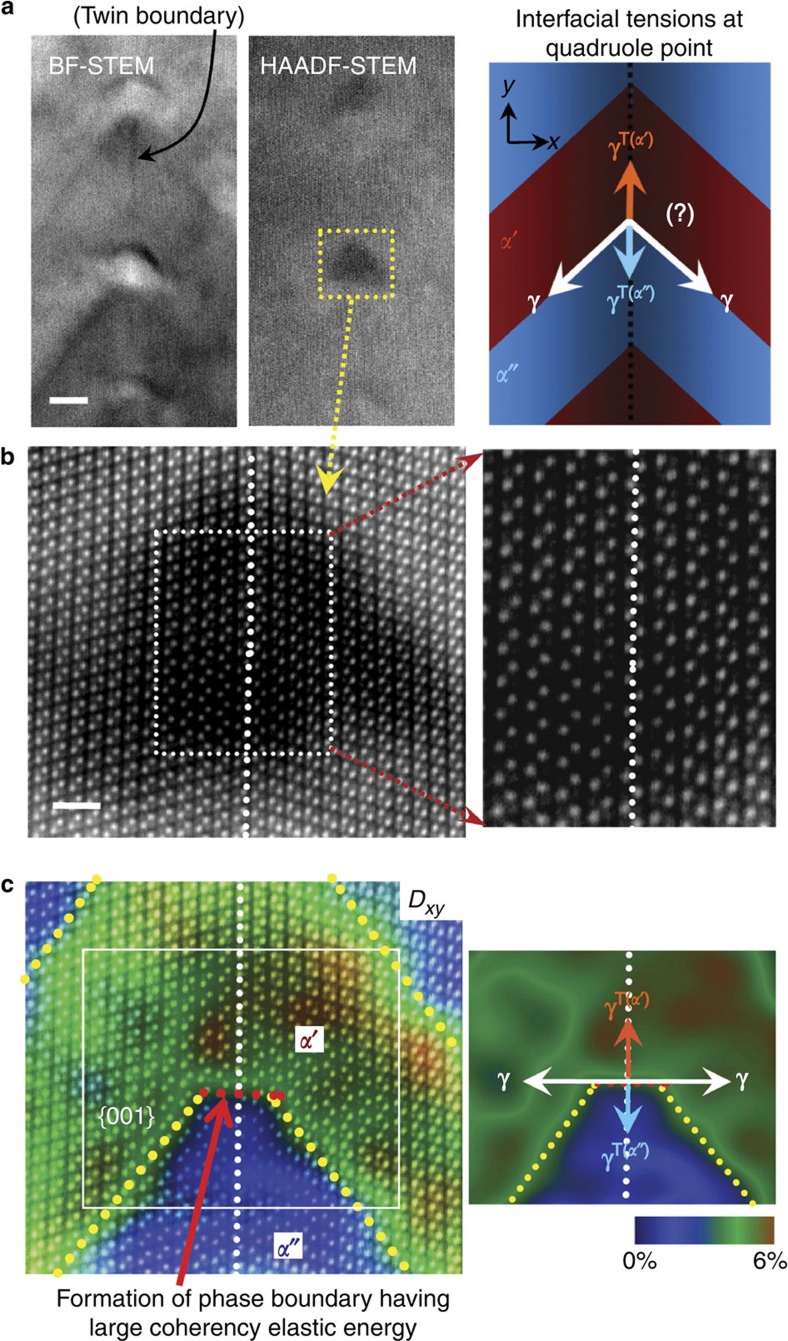
Atomic-column images at a quadruple junction and GPA for phase discrimination. (**a**) The intersection regions at the twin boundary in a quenched specimen are shown in both BF- and HAADF-STEM. The relationship between four interfacial tensions at a quadruple point is schematically depicted, based on the macroscopic Λ-shape morphology across the twin boundary. Scale bar, 5 nm. (**b**) An atomic-resolution HAADF-STEM image is demonstrated for the junction region denoted by a yellow rectangle in **a**. The magnification (right) clarifies that the quadruple junction is completely coherent without any misfit dislocations. Scale bar, 1 nm. (**c**) Formation of a phase boundary with a different orientation (red dotted line) is identified on the *D*_*xy*_ map (left) obtained from the GPA, showing bending of the {011} phase boundaries over several atomic columns. As a result, force balance between the four interfacial tensions can be achieved (right).

To scrutinize the black-contrast regions with atomic-column resolution, HAADF-STEM imaging was carried out. As electrons incoherently scattered at high angles are used in this imaging mode, the strain contrast that appeared in BF-STEM and HRTEM modes can be considerably alleviated, as seen from the comparison in Fig. 3a. Figure 3b shows a magnified HAADF-STEM image for the location denoted by a yellow rectangle in Fig. 3a. Although the strain-induced black contrast was not entirely eliminated, each atomic column was clearly observed without interference of the local strain. In particular, the enlargement on the right panel of Fig. 3b clarifies the complete lattice coherency over the intersection region. The position of phase boundaries in the HAADF-STEM image were also determined via the GPA, as already demonstrated in Fig. 1e (also see Supplementary Note 4 and Supplementary Figs 6 and 7 for utilization of GPA and variation of the *D*_*xy*_ in phase *α*′ near the quadruple junction). Figure 3c in colour is a two-dimensional map visualizing the relative lattice shear displacement obtained from the GPA. As denoted by yellow dotted lines in this map, the *α*′/*α*″ phase boundaries are distinguishable along the {001} plane. In striking contrast, the orientation of the phase boundary becomes nearly perpendicular to the twin interface around the intersection (∼2 nm in width), as indicated by a red dotted line.

When more than three interfaces are engaged at a static junction, their tension forces should be in equilibrium to satisfy the force balance. Recent observations of the deformation of an elastic substrate at a three-phase contact line with a liquid drop are noticeable results exemplifying the interfacial tension equilibrium at a junction^34^,^35^,^36^. The schematic diagram shown in Fig. 3a illustrates the relationship between four interfacial tensions at a quadruple junction under an assumption of invariant {001} phase-boundary planes. As easily recognized from this diagram, three tension components in the −*y* direction cannot be equilibrated with only one +*y* direction tension from the *α*′-phase twin boundary, *γ*^*T*(*α*′)^, unless *γ*^*T*(*α*′)^ is much larger than the remaining three tensions. Based on the similar crystal structure and the same Li_*x*_FePO_4_ compositional framework between phases *α*′ and *α*″, however, the two twin-boundary tensions, *γ*^*T*(*α*′)^ and *γ*^*T*(*α*″)^, are likely to have analogous magnitudes. Even if torque terms are taken into account with an assumption of strong inclination dependency of each boundary (see Supplementary Note 5 along with Supplementary Fig. 8 for details on Herring's relations^37^ of the force balance including torques at a junction), the force equilibrium at a quadruple junction is not attainable without change of the phase-boundary orientation. The schematic interface tension diagram drawn on the displacement map (right) in Fig. 3c demonstrates the quadruple-junction force balance achieved by considerable bending of the phase boundary around the intersection.

As already revealed in Figs 1 and 2, the present two-phase system shows anisotropy in the coherency strain energy, resulting in the majority of phase boundaries being on the {001} plane as the minimum strain energy configuration. The formation of coherent phase boundaries that are not aligned on the {001} plane therefore inevitably induces large coherency elastic energies. Figure 3c shows that the angle between the yellow and red boundaries is ∼51°, indicating a remarkably large angular deviation from the low-energy {001} plane (see Supplementary Note 6 along with Supplementary Fig. 9 for a schematic explanation). As a result, the periodic appearance of the black strain contrast at the intersections shown in Fig. 2d is now consistently understood on the basis of the locally induced large coherency elastic energy around the red phase boundary. The absence of strong image contrast at the neighbouring intersections is also noted in the STEM images. This implies that the coherency strain field confined to these junctions is comparatively lower than that of the junctions showing the black contrast.

### Comparison of two-phase morphologies

**Figure 4:**
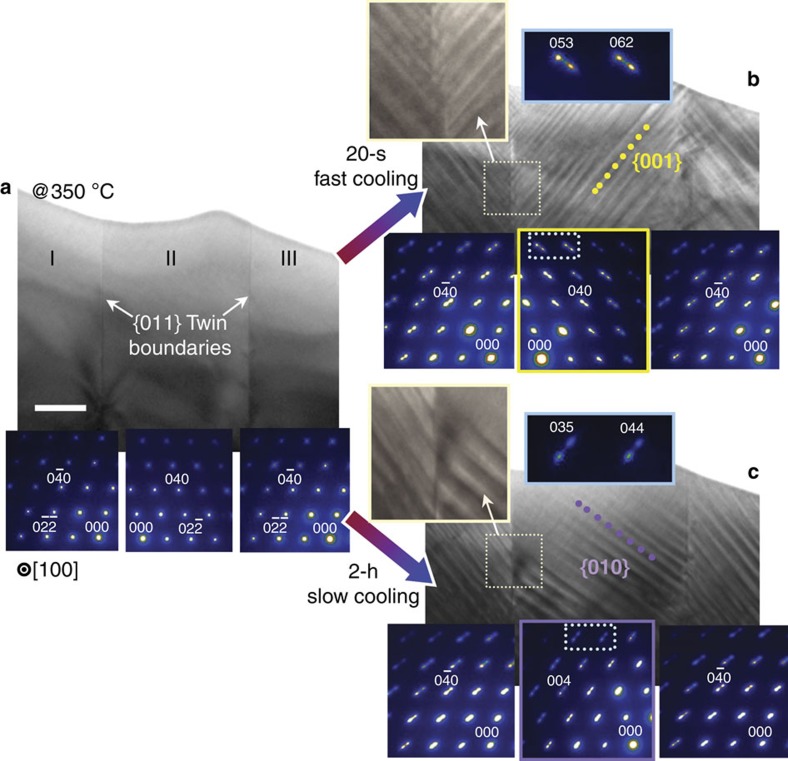
Comparison of two-phase morphologies with different cooling rates. (**a**) The grain shown in the TEM image is of a single phase at 350 °C. The presence of two parallel twin boundaries (white arrows) in the grain is verified by the mirror symmetry between the electron diffraction patterns of regions I, II and III. Scale bar, 100 nm. (**b**,**c**) Two distinct *α*′/*α*″ phase morphologies are revealed. As highlighted in the magnified images, in contrast to the V-shape phase-separation morphology after fast cooling (**b**), an asymmetrical morphology across a twin boundary is obtained when this grain is cooled slowly for 2 h (**c**). The formation of new {010} phase boundaries (red dotted line) in region II in **c** is noted (also see the enlarged diffraction patterns for each case).

We also carried out corresponding *in-situ* observations in TEM with a single grain containing two parallel twin boundaries. The BF-TEM image along with three electron diffraction patterns in Fig. 4a confirms the presence of {011} double twin boundaries in a solid-solution single phase at 350 °C. When this twinned grain was rapidly cooled from 350 °C to room temperature for 20 s, a phase-separation microstructure that is identical to that seen in Fig. 2b was consistently obtained, showing consecutive V- and Λ-shape symmetrical stripe morphologies across each of the twin boundaries (Fig. 4b). As can be verified in the electron diffraction patterns, the phase boundaries are parallel to the {001} plane. In contrast to this observation, a comprehensively different phase morphology was observed in region II, when the grain was cooled slowly to room temperature for 2 h (∼0.05 °C s^−1^ in cooling rate) after annealing the specimen again at 350 °C for 1 h to obtain a single *α*-phase. A comparison of the two BF-TEM images in Fig. 4b,c directly reveals that {010} phase boundaries have emerged in region II during the slow cooling instead of {001} boundaries. As clarified in the insets, the different split direction of the high-index Bragg spots (for example, (053) and (062) reflections in Fig. 4b, and (035) and (044) reflections in Fig. 4c) in each diffraction pattern also confirms the two distinct {001} and {010} orientations of phase boundaries depending on the cooling rate.

Quenching a TEM specimen to room temperature, far below the coherent spinodal curve in the phase diagram of Fig. 1a, causes the single *α*-phase to spinodally decompose into *α*′ and *α*′′ very rapidly, thereby resulting in the stripe morphology, as shown in Figs 2b and 4b. Phase separation via spinodal decomposition is basically achieved by compositional fluctuation with no nucleation barrier. Therefore, two-phase stripes having a {001} boundary orientation of the minimum coherency elastic energy should immediately form over an entire grain during quenching. As a result, bending of phase boundaries and subsequent high coherency strain are unavoidably induced at the quadruple junctions to satisfy the local force balance between twin and phase boundaries.

**Figure 5:**
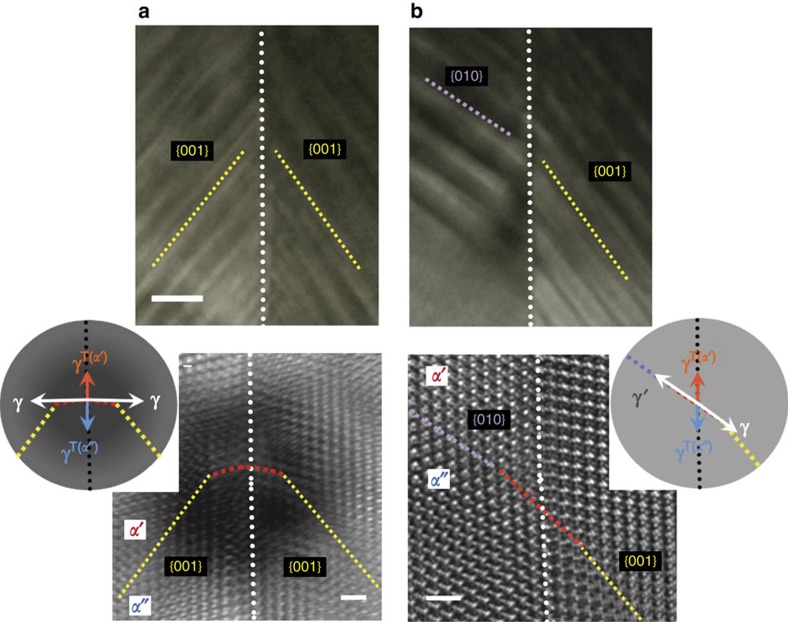
Interfacial tension equilibriums and formation of new {010} phase boundaries for coherency strain relaxation at quadruple junctions. (**a**,**b**) Along with HRTEM images of each quadruple junction, interfacial tension equilibriums are schematically depicted for both cases of quenching (**a**) and slow cooling (**b**). *γ*^*T*(*α*′)^, *γ*^*T*(*α*″)^, *γ* and *γ*′ represent the twin-interface tension in *α*′ and *α*″ phases, and the phase boundaries of the {001} and {010} planes, respectively. The presence of locally confined large coherency strain due to the red phase boundary for force equilibrium is identified with black contrast around the junction in the HRTEM image in **a**. Such a local strain field is substantially relaxed by the formation of metastable {010} phase boundaries (purple) in (**b**). Scale bar, 50 nm (upper panel) and 1 nm (lower panel).

Two sets of BF-TEM and HRTEM images together with schematic illustrations in Fig. 5 consistently explain the close relationship between interfacial tension equilibriums and the formation of new {010} phase boundaries for coherency strain relaxation at quadruple junctions. In contrast to the case where a specimen is quenched, there is sufficient time to relax any locally induced strain energies during the slow cooling for 2 h. As seen from a direct comparison of the HRTEM images of Fig. 5, this black image feature appearing at a quadruple junction in the quenched sample (Fig. 5a) is not observed in the slowly cooled sample (Fig. 5b); this reveals the absence of locally confined strain and instead demonstrates the formation of a new phase boundary with the {010} plane. Even though the {010} phase boundaries are not of the lowest coherency elastic energy, our experimental observations indicate that the formation of {010} boundaries without high lattice strain around the quadruple intersections is energetically much more favourable in the overall system. The position of phase boundaries at the junction region in the case of slow cooling was also determined via a GPA of an HAADF-STEM image (see Supplementary Fig. 10).

## Discussion

Each of the schematic illustrations in Fig. 5 describes the tangential direction of four interface tensions at the quadruple point. The tangential force balance is readily achieved with only a small amount of bending (<5°) of the {010} and {001} boundaries in the case of slow cooling (Fig. 5b), whereas substantial local bending of the {001} phase boundaries (a red dotted line) at a large angle (∼51°) from the {001} plane is necessary to meet the local force equilibrium in the case of quenching (Fig. 5a) (see Supplementary Note 7 along with Supplementary Fig. 11 for a detailed comparison). The *in-situ* experiments in Fig. 4 and the comparative quadruple-junction descriptions in Fig. 5 now allow us to reasonably understand why some of the phase boundaries (∼36%) in the furnace-cooled sample were observed to have a {010} orientation, as already noted in the pie diagram of Fig. 1d. During the sluggish furnace-cooling, {010} phase boundaries inevitably form across every twin interface as a metastable configuration to avoid the high coherency strain locally confined around the quadruple points at twin boundaries. A TEM image with a wide field of view and HRTEM images are provided in Supplementary Fig. 12, demonstrating three parallel twin boundaries and the appearance of {010} phase boundaries in a single grain of a furnace-cooled sample. An extra set of BF-TEM and HRTEM images is also shown in Supplementary Fig. 13 for a more comprehensive understanding of the distinct {010} phase-boundary formation that is not attainable without crystal twinning and local strain relaxation.

The results in this study have significant implications regarding the influence of quadruple intersections on the phase separation behaviour. First, whether metastable phase boundaries of a new orientation form in addition to the boundaries having minimum coherency elastic energy can be determined by the relative energy stability between the quadruple junctions and the newly formed phase boundaries. In contrast to our observations, for example, such new phase boundaries are not expected to form if their total coherency strain energy is relatively larger than the elastic strain energy locally confined at the quadruple points. Second, if geometrically the phase boundaries with minimum coherency elastic energy are nearly perpendicular to a twin boundary, substantial bending of the boundaries at the quadruple junctions is not necessary for the force equilibrium.

Although our study focuses on a single material system, the present experimental observations highlight the impact of lattice coherency at nanoscale quadruple-junction regions on the final macroscopic phase morphologies. In particular, crystal twinning, which involves twin boundaries, commonly occurring two-dimensional lattice defects, can be effectively used to induce completely new phase morphologies that are unachievable without twin boundaries and subsequent quadruple intersections. The local strain relaxation through the new phase boundary formation is based on the thermodynamic force equilibrium at the quadruple junction. We thus believe that such metastable phase-separation morphologies generally appear by twinning in other crystalline systems having a coherent miscibility gap, even if they are not the minimum strain energy configurations.

## Methods

### Sample preparation

Li_0.75_FePO_4_ precursor powder was first synthesized via a solid-state reaction using high-purity lithium carbonate (Li_2_CO_3_, Aldrich), iron oxalate dihydrate (Fe(II)C_2_O_4_·2H_2_O, Aldrich) and ammonium dihydrogenphosphate (NH_4_H_2_PO_4_, Aldrich). A small amount (2 mol%) of potassium carbonate (K_2_CO_3_, Aldrich) was also added as a flux. Powder mixtures of the starting materials (Li:Fe:PO_4_=0.75:1:1) with the additive were ball-milled in acetone for 24 h with zirconia milling media. A dried slurry was calcined at 350 °C for 5 h under a flow of high-purity Ar (99.999%, 400 s.c.c.m.) to prepare an amorphous Li_0.75_FePO_4_ precursor. Dense pellets with this calcined precursor were sintered at 800 °C for 3 h in the same Ar atmosphere to finally obtain polycrystalline samples for *in-situ* experiments. TEM specimens were prepared by mechanical grinding to a thickness of 80 μm, followed by dimpling to a thickness of <10 μm and ion-beam thinning for electron transparency.

### Transmission electron microscopy

Conventional HRTEM images were acquired using a TEM (JEM-2100F, JEOL) operated at 200 kV. BF-STEM and HAADF-STEM images were also obtained using the same electron microscope with a spherical aberration corrector (CEOS GmbH) for probe-forming lenses. The size of the electron probe in STEM mode was 0.96 Å with a convergence semiangle of 22 mrad. The collection semiangles of the HAADF detector were adjusted from 71 to 190 mrad, to use incoherently scattered electrons at large angles for clear *Z*-sensitive images. For *in-situ* observations during heating and after quenching, a hot-stage heating holder (Model 652, Gatan)^38^,^39^,^40^,^41^ was used in a TEM capable of a high specimen tilt (JEM-2100, JEOL). The heating system based on a ring-type furnace made from tantalum in the holder enables homogeneous and rapid heating up to 1,000 °C. Cooling from 350 °C to room temperature could be achieved in ∼10 s by turning off the furnace in the holder.

### X-ray diffraction and GPA

For macroscopic phase identification, X-ray diffraction patterns were collected in a diffractometer (D/max2500, Rigaku) at 40 kV and 120 mA using Cu-K_α_ radiation. A GPA with HAADF-STEM images was carried out using GPA Phase (HREM Research Inc.). As the two-dimensional displacement field relative to the reference net in the image can be obtained from a set of noncolinear Fourier components in this analysis, the *α*′/*α*″ phase distribution and resulting position of phase boundaries are efficiently visualized. Details of using the GPA are provided in Supplementary Notes 2 and 4.

## Additional information

**How to cite this article:** Chung, S.-Y. *et al.* Quadruple-junction lattice coherency and phase separation in a binary-phase system. *Nat. Commun.* 6:8252 doi: 10.1038/ncomms9252 (2015).

## Supplementary Material

Supplementary Information Supplementary Figures 1-13, Supplementary Notes 1-7 and Supplementary References
