# Supplementary material for: Quadruple-junction lattice coherency and phase separation in a binary-phase system
Source: Nat Commun. 2015 Sep 8;6:8252. doi: 10.1038/ncomms9252 (PMC4569863; doi:10.1038/ncomms9252)
Supplement: Supplementary Information — Supplementary Figures 1-13, Supplementary Notes 1-7 and Supplementary References [file ncomms9252-s1.pdf]

## Supplementary Information

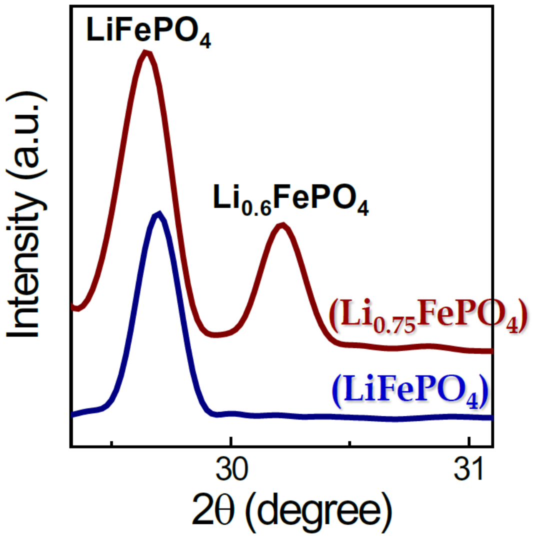


**Supplementary Figure 1. X-ray diffraction patterns.** In contrast to the single (211)/(020) peak for $LiFePO_4$ in the blue pattern, two (211)/(020) major peaks appear in the red pattern, verifying the $LiFePO_4$/$Li_{0.6}FePO_4$ two-phase mixture (see the powder diffraction data from a previous report by Chen *et al.*[1] for comparison). Our samples for the X-ray diffraction were prepared by sintering at 800°C for 3 h in Ar followed by furnace-cooling for 8 h.

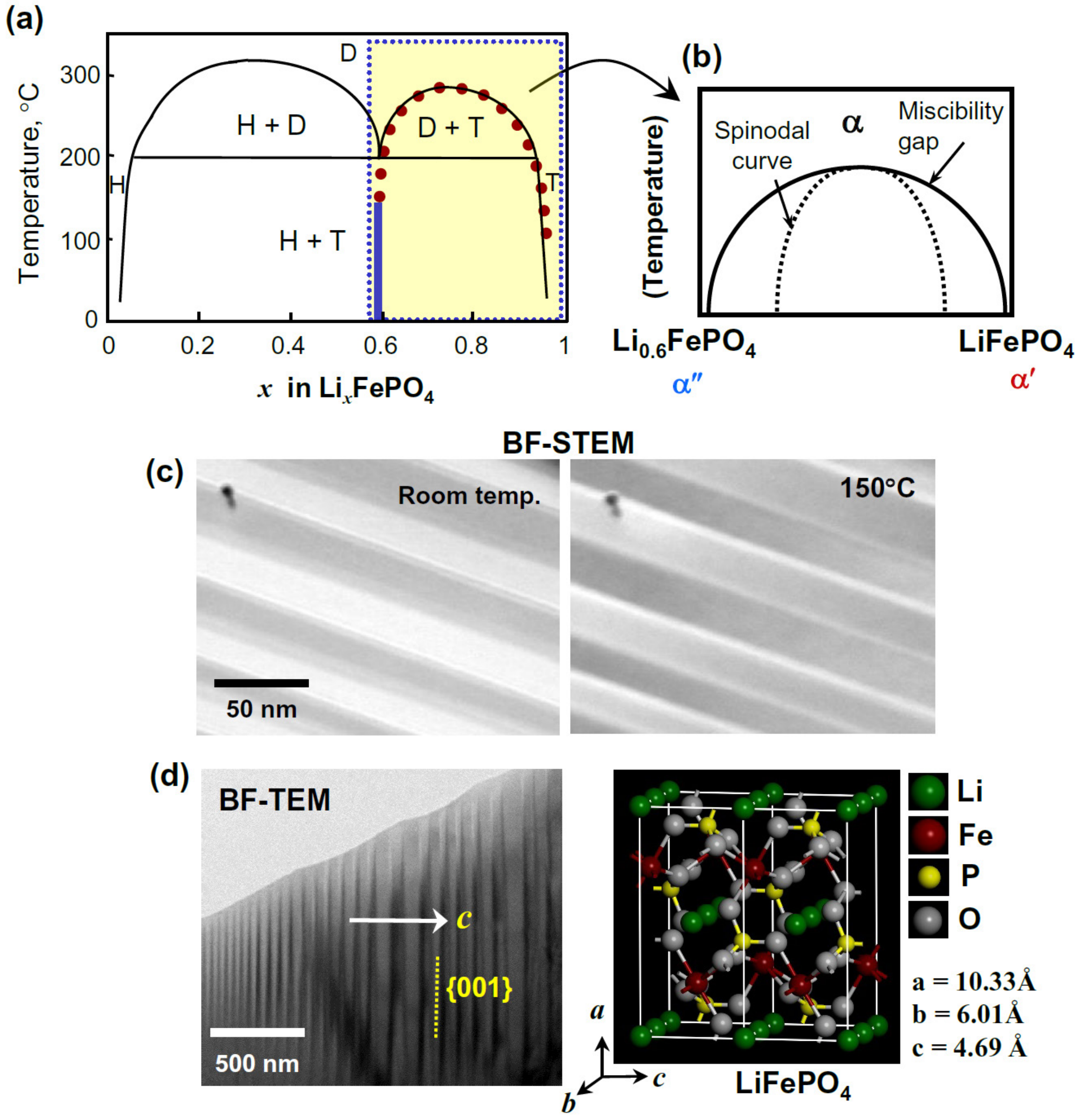


**Supplementary Figure 2. Phase diagrams of Li$_x$FePO$_4$ and two-phase TEM images.** (**a**) This diagram is based on a previous study by Dodd *et al.*[2]. T, H, and D in the phase diagram indicate LiFePO$_4$ (triphylite), FePO$_4$ (heterosite), and disordered Li$_x$FePO$_4$. The thick blue line denotes the position of the intermediate Li$_{0.6}$FePO$_4$ phase. (**b**) A schematic phase diagram between LiFePO$_4$ and metastable Li$_{0.6}$FePO$_4$ is illustrated. The broken line inside the miscibility gap represents phase separation by spinodal decomposition. (**c**) The left image was taken from a grain at room temperature after furnace-cooling. This two-phase stripe morphology with {001} phase boundaries does not vary during annealing at 150°C (right image), although the black/white stripe image contrast was inverted in BF mode due to the thermal agitation. (**d**) This BF-TEM image shows that the phase boundaries have the {001} orientation. The unit-cell structure of orthorhombic LiFePO$_4$ is also illustrated in the right panel.

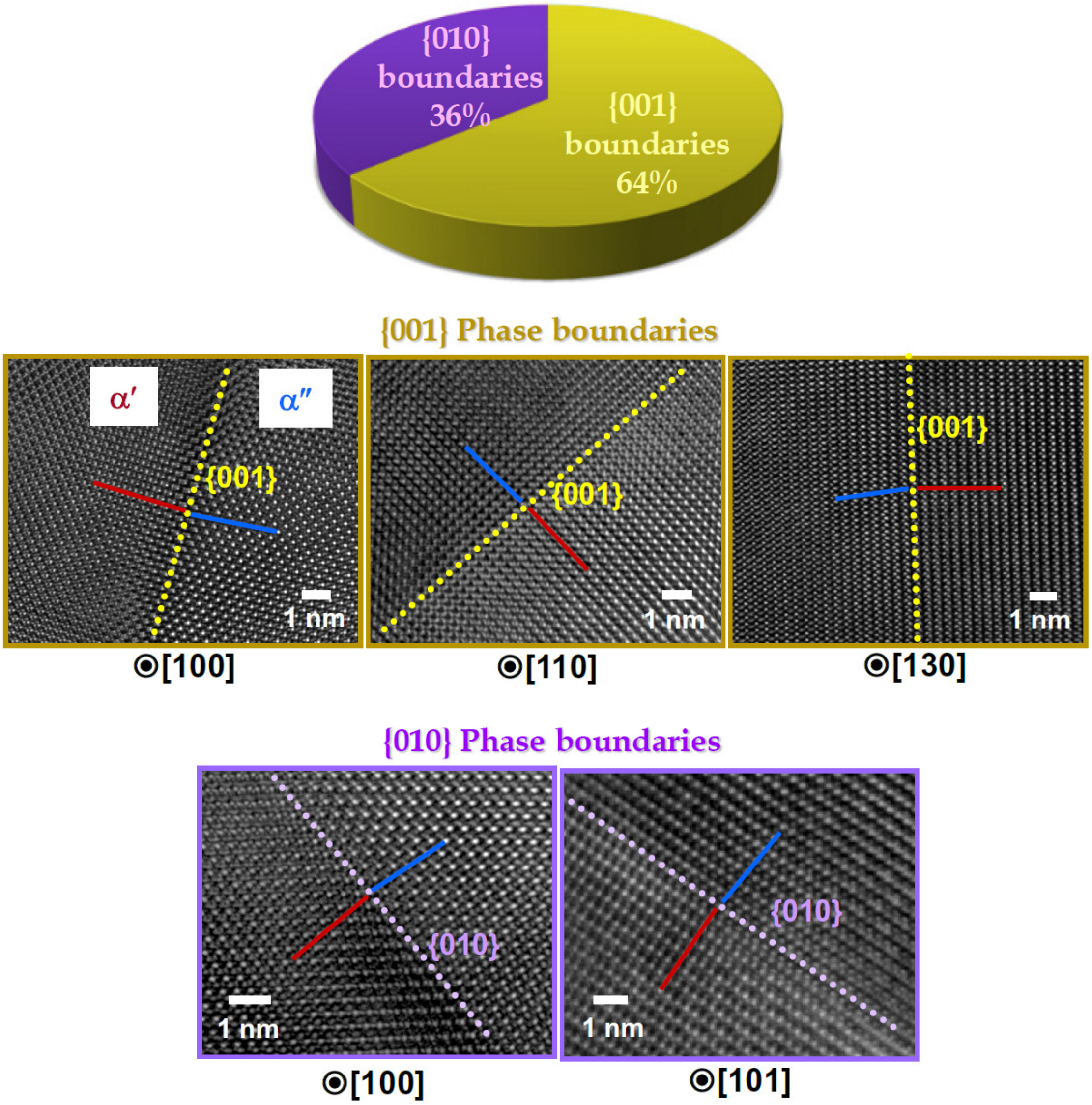


**Supplementary Figure 3. Types of α′/α″ phase boundaries.** {001} and {010} phase boundaries are identified during the investigation of more than 40 grains in a furnace-cooled specimen. A pie diagram shows the statistical result, indicating that the majority of phase boundaries are along the {001} plane. As exemplified in each of the HRTEM images, the phase boundaries are coherent.

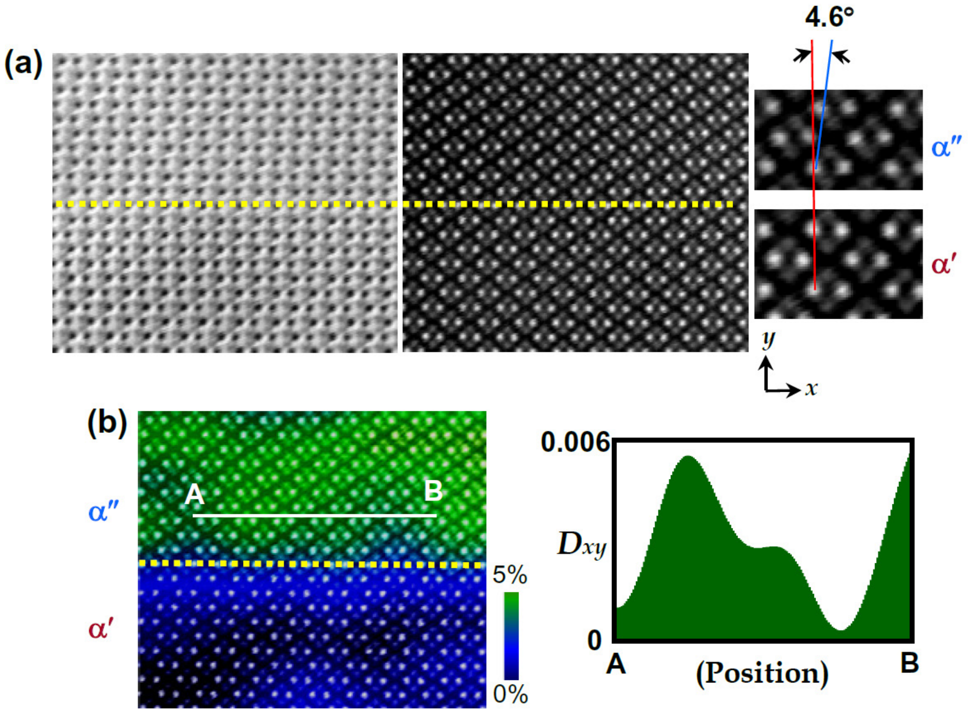


**Supplementary Figure 4. GPA for phase discrimination in a STEM image.** (**a**) BF-STEM (left) and corresponding HAADF-STEM (middle) images show the completely coherent phase boundary (yellow line). In the enlargements (right), the difference in the axial angle between phases α′ and α″ is noted. (**b**) The left color map demonstrates the $D_{xy}$ result from the GPA, showing ~5% variation in the shear displacement between the two phases. The right profile between locations A and B in the image exemplifies that the $D_{xy}$ deviation caused by e-beam scanning and specimen drift does not exceed 0.6%.

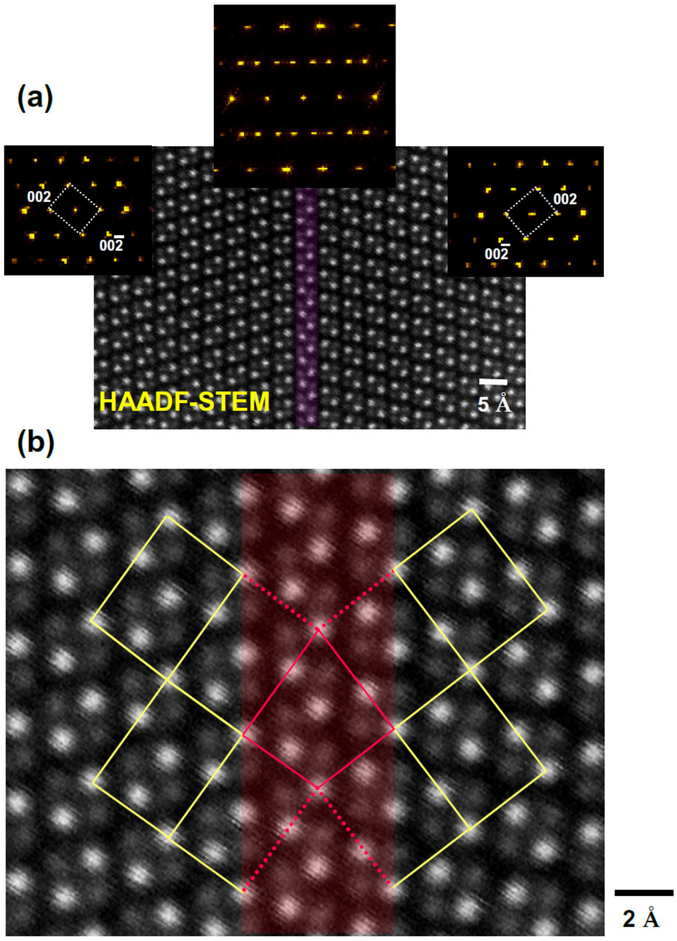


**Supplementary Figure 5. HAADF-STEM images showing an atomic configuration of a twin boundary.** (**a**) FFTs on each of the sides represent a typical twin relationship across a boundary denoted in purple in the image. The FFT in the center is obtained from the both twinss. (**b**) In this magnified atomic-column image, the inverted mirror reflection across the twin boundary can be recognized. Each unit cell is denoted by a yellow rectangle. A periodic atomic unit at the boundary region (red shadow) is also indicated by red lines.

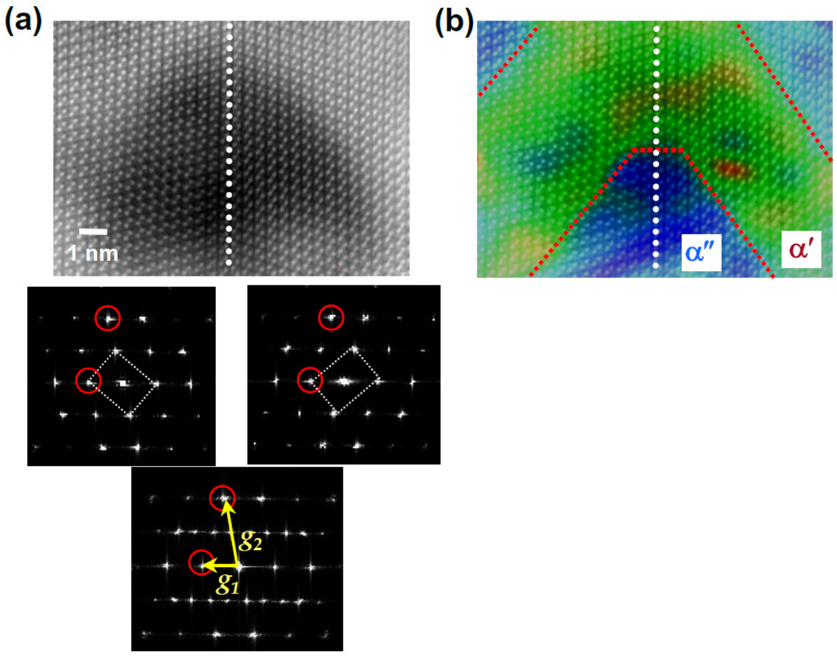


**Supplementary Figure 6. GPA in an image with a twin boundary.** (**a**) This HAADF-STEM image was taken from a quenched specimen. Small red circles in each of the FFTs indicate the Bragg spots common to both twins. (**b**) The color map shows the $D_{xy}$ result obtained from $\boldsymbol{g_1}$ and $\boldsymbol{g_2}$, as indicated by yellow arrows in (**a**). Red broken lines denote the phase boundaries, revealing the bending of boundaries at the quadruple junction.

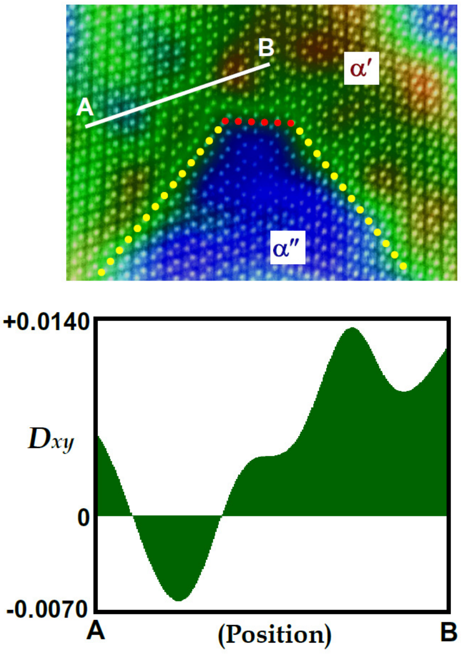


**Supplementary Figure 7. Variation of the shear displacement (*$D_{xy}$*) near the quadruple junction.** As shown in the $D_{xy}$ map superimposed on the HAADF-STEM image, relatively high coherency strain is locally confined near the quadruple junction. However, as the $D_{xy}$ variation induced by the coherency strain within phase α′ is much smaller (less than 2%) than the difference in $D_{xy}$ (~5%) between phases α′ and α″, the phase boundaries can be readily identified in the GPA map. The $D_{xy}$ profile between locations A and B is plotted, verifying that the maximum variation is <2%. Therefore, the GPA can be efficiently utilized for determining the phase-boundary position in STEM images.

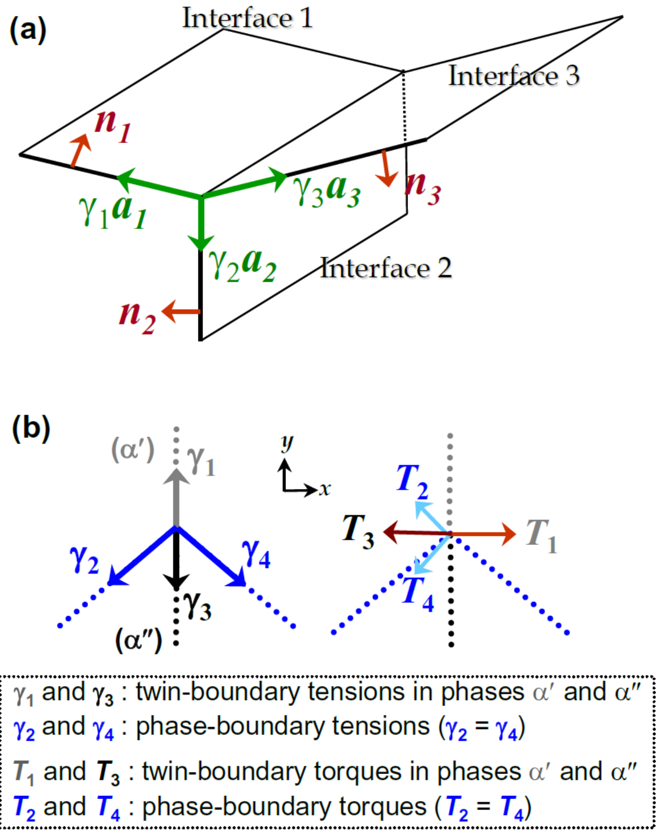


**Supplementary Figure 8. Force diagrams at multiple junctions.** (**a**) Force equilibrium at a triple junction is illustrated. In addition to the three tensions, torque terms can contribute to the total force balance in a case where the tension values of the three interfaces have very strong dependency on inclination. The torque of each interface is exerted along the interface normal, as denoted by $\boldsymbol{n_1}$, $\boldsymbol{n_2}$, and $\boldsymbol{n_3}$, respectively. (**b**) These diagrams illustrate the quadruple junction in our study without consideration of phase-boundary bending, as already shown in the schematic illustration of Fig. 3a in the main text. Each diagram depicts the directions of four tensions (left) and of four torques (right) exerted upon the junction. The presence of unbalanced force components can be identified in both of the diagrams, demonstrating that this junction is not at equilibrium.

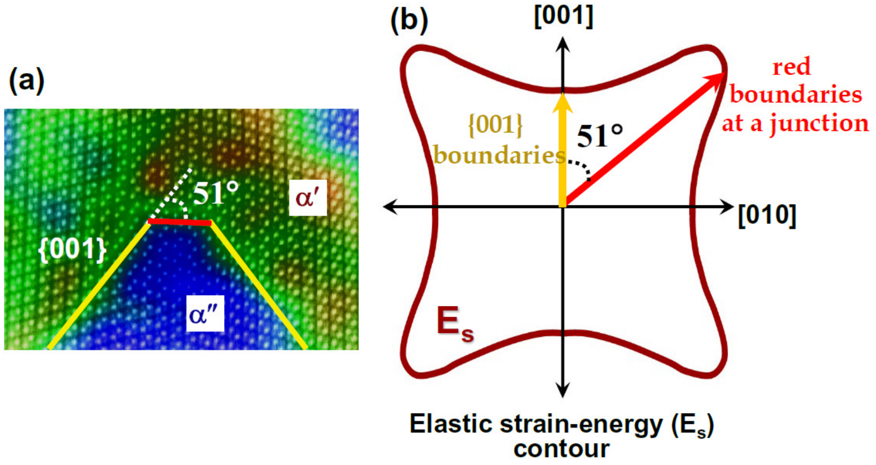


**Supplementary Figure 9. Orientation angle difference between phase boundaries and their relative coherency elastic strain energies.** (**a**) A phase boundary with a different orientation at an angle of ~51° from the {001} plane is denoted in red. (**b**) In this diagram, comparatively larger coherent strain energy is shown for the red boundary.

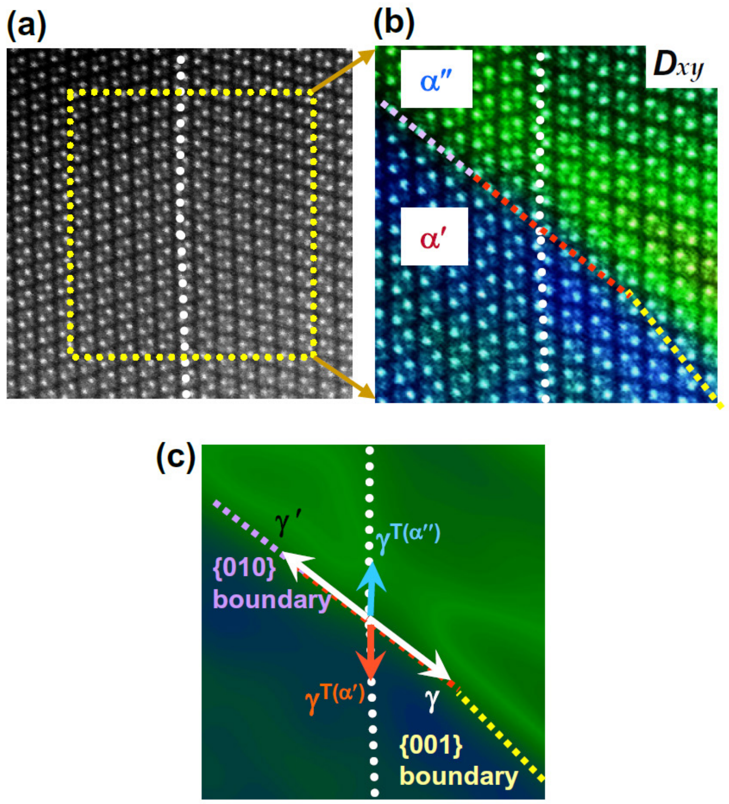


**Supplementary Figure 10. Phase discrimination at a quadruple intersection via GPA.** (**a**) The atomic-column HAADF-STEM image (right) was taken from a quadruple-junction region in a slowly cooled specimen. The white dotted line indicates the twin boundary. (**b**) The relative shear displacement ($D_{xy}$) map is shown in color, distinguishing the α′ and α″ phases. The resulting phase boundary is denoted by yellow and red lines. (**c**) The interfacial force equilibrium is schematically described at the quadruple junction. $\gamma^{T(\alpha')}$, $\gamma^{T(\alpha'')}$, γ, and γ′ represent the twin-interface tension in α′ and α″ phases and the phase boundaries of the {001} and {010} planes, respectively.

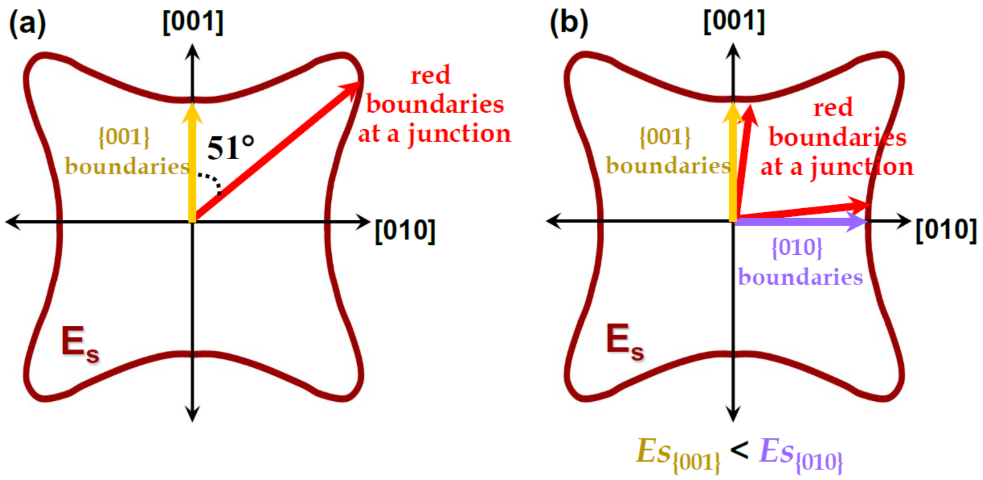


**Supplementary Figure 11. Coherency strain energy comparison.** (**a**) Fast quenching case. The red phase boundaries, which considerably deviate from the {001} orientation, induce relatively high coherency strain. (**b**) Slow cooling case. Although the coherency strain energy of the {010} boundaries is somewhat larger that that of the {001} boundaries, there is no high strain locally concentrated near the junctions, leading to an energetically more stable configuration.

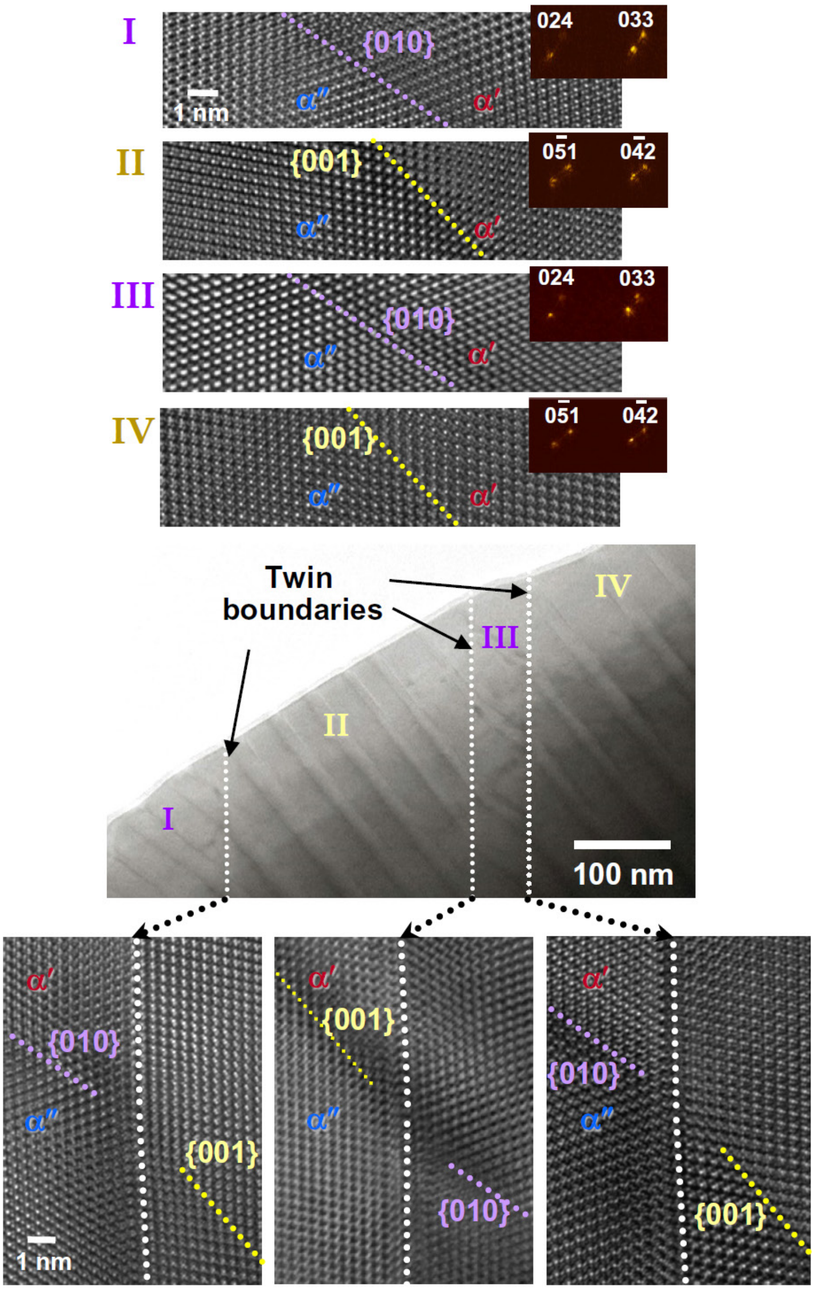


**Supplementary Figure 12. Wide field-of-view TEM and HRTEM images.** This set of images shows the phase boundaries and the quadruple intersections in a furnace-cooled specimen. The low-magnification image in the center provides a wide view of a grain having three parallel twin boundaries (white dotted lines). As specifically demonstrated in the HRTEM images in the upper panel, {001} phase boundaries form in regions II and IV, while {010} phase boundaries appear in regions I and III. Therefore, asymmetrical stripe morphologies, which are identical to those in Fig. 4c in the main text, are observed across each of the twin boundaries. The three HRTEM images in the lower panel consistently demonstrate the coherent lattice without local strain-induced black contrast at quadruple junctions along the twin boundaries.

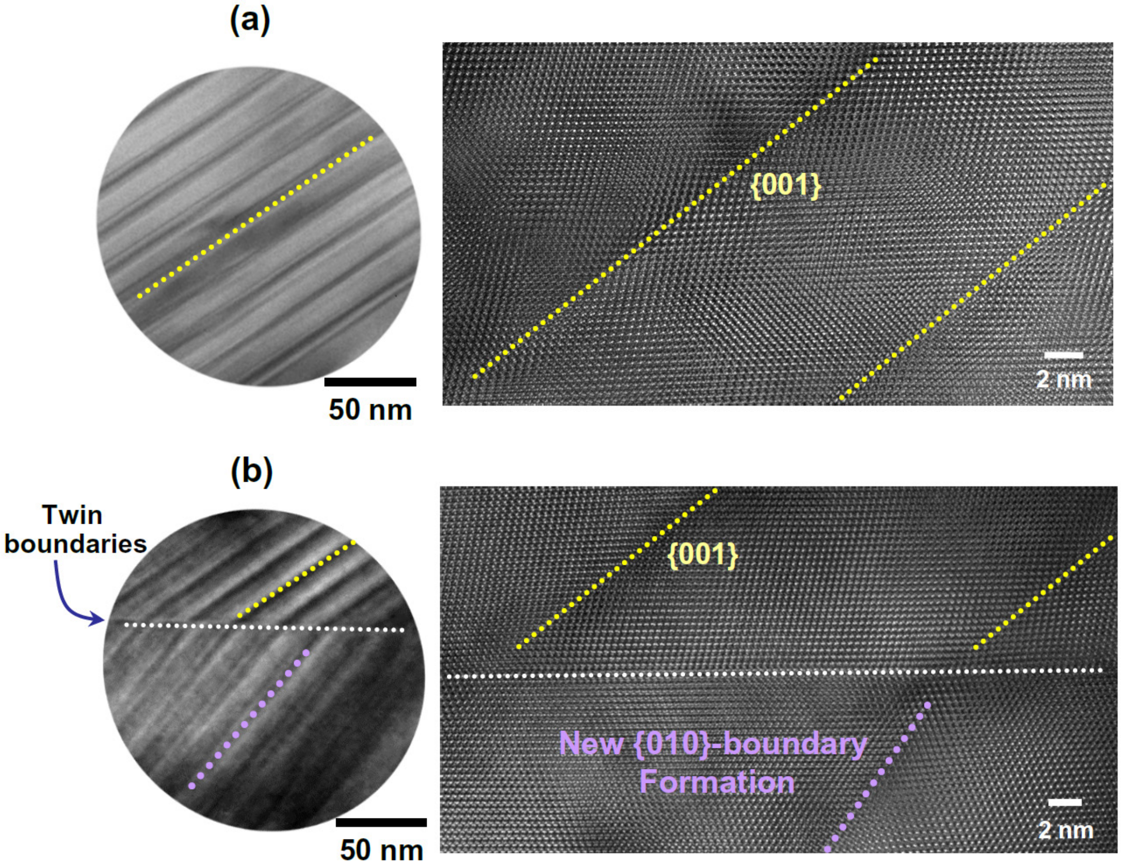


**Supplementary Figure 13. Extra set of BF-TEM and HRTEM images for phase separation morphology.** These images clearly show that the overall phase morphology varies considerably by crystal twinning. (**a**) Without a twin boundary, the lamellar-type phase separation takes place in every single grain along with the formation of {001} phase boundaries (yellow lines) as the lowest coherency strain-energy configuration. Therefore, such an energetically stable orientation of the {001} phase boundaries is not affected by any kinetic parameters including annealing and subsequent cooling rates. (**b**) As can be seen in Fig. 5 in the main text, the high coherency strain is inevitably induced around the quadruple junctions to satisfy the force balance condition when a twin boundary intersects with the {001} phase boundaries in a grain. If sufficient annealing time is given, the locally high strain field can be substantially relaxed by the formation of metastable {010} phase boundaries (purple lines), resulting in a distinct phase-separation morphology that is not attainable without crystal twinning.

**Supplementary Note 1.** Phase diagram between $LiFePO_4$ and $Li_{0.6}FePO_4$

On the basis of electrochemical delithiation reactions studied since 1997, many reports have shown that there is a miscibility gap between $LiFePO_4$ and $FePO_4$ at room temperature, demonstrating a well-known two-phase reaction during electrochemical cycling in $LiFePO_4$ (refs 1–4). In 2007, however, Chen *et al.* systematically proved the presence of two intermediate metastable phases, $Li_{0.6}FePO_4$ and $Li_{0.34}FePO_4$, below 200°C in the $LiFePO_4/FePO_4$ system through high-temperature *in situ* X-ray diffraction[1]. In particular, they clarified the stability of the $Li_{0.6}FePO_4$ phase even after 5 months, noting that neither very slow cooling nor long-term annealing at 100°C stimulated further phase change.

Samples used in this study were also verified to have a $LiFePO_4/Li_{0.6}FePO_4$ two-phase mixture. As compared with single-phase $LiFePO_4$ (blue pattern) in the X-ray diffraction of Supplementary Fig. 1, our specimen having an initial composition of $Li_{0.75}FePO_4$ (red pattern) clearly shows an additional (211)/(020) major peak for $Li_{0.6}FePO_4$, in good agreement with the previous report.

Based on X-ray powder diffraction in 2006, Dodd *et al.* provided a phase diagram of $Li_xFePO_4$ ($0 \leq x \leq 1$) in the range of 180–380°C (see Supplementary Fig. 2a)[2]. Because their work largely focused on the high-temperature regions above 180°C, as indicated by the data points in the phase diagram, they did not make any specific remarks on the presence of intermediate phases. However, the studies Dodd *et al.*[2] and Chen *et al.*[1] taken together offer a plausible phase diagram extended to room temperature between $LiFePO_4$ ($\alpha'$) and $Li_{0.6}FePO_4$ ($\alpha''$), as denoted by a red dotted curve in the yellow shade of Supplementary Fig. 2a. Furthermore, a recent report by Furutsuki *et al.*[5] also confirmed the presence of the intermediate $Li_{0.6}FePO_4$ phase. Supplementary Fig. 2b schematically illustrates the resultant $\alpha'/\alpha''$ phase diagram, presenting a miscibility dome and a spinodal curve. During annealing of our two-phase specimen at 150°C, no substantial change in the stripe morphology could be observed, as seen also in the BF-STEM images of Supplementary Fig. 2c. Therefore, the $LiFePO_4/Li_{0.6}FePO_4$ immiscible dome ranging from room temperature to ~280°C is reasonably supported.

The difference in each of the lattice parameters between $LiFePO_4$ and $Li_{0.6}FePO_4$ is summarized below on the basis of the information provided in the previous report[1].

$$LiFePO_4 : a = 10.33\ \text{Å},\ b = 6.01\ \text{Å},\ c = 4.69\ \text{Å}$$
$$Li_{0.6}FePO_4 : a = 10.20\ \text{Å},\ b = 5.93\ \text{Å},\ c = 4.73\ \text{Å}$$
$$\Delta a = -1.16\%,\quad \Delta b = -1.31\%,\quad \Delta c = +0.85\%$$

As can be seen, differences of less than ±1.5% are identified. Consequently, the formation of coherent phase boundaries is readily anticipated during temperature-driven phase separation. The unit cell of orthorhombic $LiFePO_4$ is also illustrated along with a BF-TEM image showing the stripe morphology in Supplementary Fig. 2d.

**Supplementary Note 2.** Utilization of geometric phase analysis (GPA) for discrimination of the α′/α″ phases on HAADF-STEM images

In addition to mapping local displacement fields from high-resolution lattice images[6,7], geometric phase analysis (GPA) has been efficiently utilized to visualize the discrimination of nanoscale domains having slightly different lattice dimensions relative to each other[8,9]. The two-dimensional displacement field, $u(\boldsymbol{r})$, is obtained from the relative phase shifts $P_{g1}(\boldsymbol{r})$ and $P_{g2}(\boldsymbol{r})$ of a particular set of noncolinear Fourier components, $\boldsymbol{g_1}$ and $\boldsymbol{g_2}$ in the image, as follows;

$$u(\boldsymbol{r}) = -\frac{1}{2\pi}[\, P_{g1}(\boldsymbol{r})\,\boldsymbol{a_1} + P_{g2}(\boldsymbol{r})\,\boldsymbol{a_2}]$$

where $\boldsymbol{a_1}$ and $\boldsymbol{a_2}$ are the lattice vectors in real space for $\boldsymbol{g_1}$ and $\boldsymbol{g_2}$, respectively. Therefore, the comparative displacements, including the uniaxial ($D_{xx}$ and $D_{yy}$) and diagonal shear ($D_{xy}$) components, with respect to a reference can be derived from the following derivatives of $u(\boldsymbol{r})$.

$$D_{xx} = \frac{\partial u_x(\boldsymbol{r})}{\partial x}, \quad D_{yy} = \frac{\partial u_y(\boldsymbol{r})}{\partial y}, \quad D_{xy} = \frac{1}{2}\left(\frac{\partial u_y(\boldsymbol{r})}{\partial x} + \frac{\partial u_x(\boldsymbol{r})}{\partial y}\right)$$

As shown in Supplementary Fig. 4a, the α′ and α″ phases in the present study are completely coherent across the phase boundaries with no substantial difference in the unit-cell dimensions (less than ±1.5% as explained in Supplementary Note 1). Consequently, the use of either $D_{xx}$ or $D_{yy}$ is not appropriate for phase discrimination and determination of a boundary position. In contrast, as the vertical axis of phase α″ inclines by 4.6° from that of phase α′ in the two-dimensional unit cell, sufficient variation in the shear component, $D_{xy}$, is provided to distinguish the two phases in STEM images. Supplementary Fig. 4b presents a $D_{xy}$ map superimposed on a HAADF-STEM image. Phases α′ and α″ (dark blue and green) and their boundary (yellow line) are straightforwardly visualized in this map. As exemplified in the plot of Supplementary Fig. 4b, the $D_{xy}$ deviation within a single phase is less than 0.6%, verifying the high accuracy of the boundary-position determination via GPA in STEM images.

**Supplementary Note 3.** Atomic structure of a {011} twin interface

In contrast to typical twins of mirror symmetry in many crystalline metals, $Li_xFePO_4$ in this study is found to have "inversion twins" during the HAADF-STEM analysis. As already reported for CdS and ZnO crystals[10,11], twins with an inverted mirror reflection are identified. Their boundaries are along the {011} plane. The specific atomic structure of a {011} twin boundary is provided in Supplementary Fig. 5. Multiple twin boundaries mutually parallel to each other also can be observed during the HRTEM analysis.

**Supplementary Note 4.** Utilization of geometric phase analysis (GPA) with HAADF-STEM images containing a twin boundary

When a GPA is carried out for images of a quadruple junction containing a twin boundary, the selection of two noncolinear Fourier components, $\boldsymbol{g_1}$ and $\boldsymbol{g_2}$, is important. To avoid the relative phase shift induced from the twin relation and extract the displacement field exclusively by the geometric variation between the $\alpha'$ and $\alpha''$ phases, it is necessary to select common Fourier components from both sides of twins. As can be seen in Supplementary Fig. 6a, the Bragg spots denoted by a red circle are common in both FFTs. Therefore, if the two reciprocal lattice vectors, $\boldsymbol{g_1}$ and $\boldsymbol{g_2}$, are taken, for example, as indicated by yellow arrows in the central FFT, the $D_{xy}$ variation in the two phases can be acquired regardless of the presence of a twin boundary, thereby enabling the $\alpha'/\alpha''$phase discrimination. Supplementary Fig. 6b shows the $D_{xy}$ map obtained from the $\boldsymbol{g_1}$ and $\boldsymbol{g_2}$ vectors in the FFT of Supplementary Fig. 6a, indicating the phase boundaries with red broken lines. Local variation of $D_{xy}$ due to the high strain field by the phase-boundary bending is also recognizable around the quadruple point in this map.

**Supplementary Note 5.** Herring's relations at a multiple junction

When multiple interfaces intersect with each other at a static junction, they should be in force equilibrium. As generally known from Young equations, the sum of the interface tensions acting on a junction should be zero for force balance. Supplementary Fig. 8a shows a triple junction where three different interfaces meet. To satisfy the local force equilibrium condition at this junction, three interfacial tensions must be balanced along their tangential direction, resulting in the following equation:

$$\gamma_1 \boldsymbol{a_1} + \gamma_2 \boldsymbol{a_2} + \gamma_3 \boldsymbol{a_3} = 0 \quad \text{or} \quad \sum_{i=1}^{3} \gamma_i \boldsymbol{a_i} = 0$$

where $\gamma_i$ is the interfacial tension of the $i$th interface and $\boldsymbol{a_i}$ is a unit vector lying in the $i$th interface.

In addition to the interface tensions, Herring in 1951 postulated the torque terms as another contribution to the total force balance[12]. Some special interfaces, for example Σ-type ordered grain boundaries, can have very strong dependency on inclination; a small change in inclination of such interfaces usually causes substantial variation in γ values, tending to twist the interfaces. Consequently, a significant amount of torque, $T_i$ = $(\partial\gamma_i/\partial\phi_i)$, where $\phi_i$ is the inclination angle of the $i$th interface, can be exerted upon the junction. As a result, the following equation, known as Herring's relations, is derived for the force balance at a multiple junction[12]:

$$\sum_{i=1}^{n}\{\gamma_i\, \boldsymbol{a_i} + \left(\frac{\partial\gamma_i}{\partial\phi_i}\right)\boldsymbol{n_i}\} = 0$$

where $\boldsymbol{n_i}$ is the unit boundary normal of the $i$th interface.

Supplementary Fig. 8b shows the present case of a quadruple junction, at which two $\alpha'/\alpha''$ phase boundaries (blue lines) and two different twin boundaries in phases $\alpha'$ (gray line) and $\alpha''$ (black line), respectively, intersect with each other. The schematic force diagrams illustrate the tensions (left) and the torques (right) of the four interfaces upon the quadruple junction without bending of the phase boundaries. As the $\alpha'/\alpha''$ phase boundaries are identical, their tensions and torques are equal to each other ($\gamma_2 = \gamma_4$ and $T_2 = T_4$). In addition, phases $\alpha'$($LiFePO_4$) and $\alpha''$($Li_{0.6}FePO_4$) have the same structural $Li_xFePO_4$ framework with a small misfit in lattice parameters. Therefore, the values of the twin-boundary tensions, $\gamma_1$ and $\gamma_3$ (and also the twin-boundary torques, $T_1$ and $T_3$), are not expected to be substantially different between $\alpha'$ and $\alpha''$. As represented in the force diagrams of Supplementary Fig. 8b, unbalanced force components both in γ along the $\pm y$ direction (left) and in $T$ along the $\pm x$ direction (right) are recognized. It is thus apparent that different configurations between the boundaries are necessary in order to achieve local force equilibrium at the junction.

Although qualitatively represented, the force diagram in Fig. 3c of the main text, which is based on direct HAADF-STEM observations, clearly shows the balance between the interfaces tensions achieved by bending of the $\alpha'/\alpha''$ phase boundaries near the quadruple junction. As described in Supplementary Fig. 8a, torque is the force normal to the interface tangential tension. Thus, even if it is assumed that the influence of torques from the four interfaces is not negligible in our case, no force components of γ and $T$ remain unbalanced against the Herring relations at the nearly cross-shaped quadruple intersection shown in Fig. 3c in the main text.

**Supplementary Note 6.** High coherency elastic strain energy at a junction

Based on the TEM observations, the elastic strain energy (*Es*) contour has been suggested, as depicted in Fig. 1d in the main text. The phase boundaries in red at a quadruple junction region in Supplementary Fig. 9a show a large deviation angle, 51°, from the {001} phase boundaries having the lowest *Es*. Although this *Es* contour is qualitative, the relatively larger coherency strain energy induced by the red phase boundaries, as indicated by a red arrow in Supplementary Fig. 9b, consistently supports the periodic presence of local black contrasts in Fig. 2d.

**Supplementary Note 7.** Comparison between quenching and slow cooling cases

As already shown in Fig. 5 in the main text, in order to meet the force balance at a quadruple intersection the formation of red phase boundaries with different orientations is inevitable. When a specimen is quenched to room temperature, the red phase boundaries at the junctions are at an angle of ~51° from the {001} plane, thereby inducing considerably high strain energy near the junctions (Supplementary Fig. 11a). In contrast, due to the formation of the new {010} phase boundaries during slow cooling, small-angle (less than 5°) bending of the red phase boundaries is necessary for force balance at the junctions. Consequently, no locally high strain energy is present near the junction regions in the slowly cooled specimen (Supplementary Fig. 11b).

## Supplementary References


1. Chen, G., Song, X. & Richardson, T. J. Metastable solid-solution phases in the $LiFePO_4/FePO_4$ system. *J. Electrochem. Soc.* **154**, A627−A632 (2007).
2. Dodd, J. L., Yazami, R. & Fultz, B. Phase diagram of $Li_xFePO_4$. *Electrochem. Soild-State Lett.* **10**, A151−A155 (2006).
3. Yamada, A., Chung, S. C. & Hinokuma, K. Optimized $LiFePO_4$ for lithium battery cathodes. *J. Electrochem. Soc.* **148**, A224–A229 (2001).
4. Meethong, N., Huang, H.-Y. S., Carter, W. C. & Chiang, Y.-M. Size-dependent lithium miscibility gap in nanoscale $Li_{1-x}FePO_4$. *Electrochem. Soild-State Lett.* **10**, A134−A138 (2007).
5. Furutsuki, S., Chung, S.-C., Nishimura, S.-I., Kudo, Y., Yamashita, K. & Yamada, A. Electrochromism of $LiFePO_4$ induced by intervalence charge transfer transition. *J. Phys. Chem. C* **116**, 15259−15264 (2012).
6. Hÿtch, M. J., Snoeck, E. & Kilaas, R. Quantitative measurement of displacement and strain fields from HREM micrographs. *Ultramicroscopy* **74**, 131−146 (1998).
7. Hÿtch, M. J. & Plamann, T. Imaging conditions for reliable measurement of displacement and strain in high-resolution electron microscopy. *Ultramicroscopy* **87**, 199–212 (2001).
8. Catalan, G. *et al.* Flexoelectric rotation of polarization in ferroelectric thin films. *Nat. Mater.* **10**, 963−967 (2011).
9. Rossell, M. D. *et al.* Atomic structure of highly strained $BiFeO_3$ thin films. *Phys. Rev. Lett.* **108**, 047601 (2012).
10. Iwanaga, H., Tomizuka, A., Takeuchi, S. & Yoshiie, T. Five types of inversion twin in vapour-grown CdS needle crystals. *Phil. Mag. A* **64**, 303–309 (1991).
11. Yan, Y. & Al-Jassim, M. M. $[1\bar{1}00]/(1102)$ twin boundaries in wurtzite ZnO and group-III nitrides. *Phys. Rev. B* **71**, 041309(R) (2005).
12. Herring, C. Surface tension as a motivation for sintering in *The Physics of Powder Metallurgy* (edited by W. E. Kingston), McGraw-Hill, New York, pp.143−178 (1951).
